# Round-Robin test for the histological diagnosis of acute colonic Graft-versus-Host disease validating established histological criteria and grading systems

**DOI:** 10.1007/s00428-023-03544-3

**Published:** 2023-05-11

**Authors:** Katrin Hippe, Andreas Kreft, Simone Reu-Hofer, Andreas Rosenwald, Fulvia Ferrazzi, Christoph Daniel, Kerstin Amann, Sabrina Kraus, Ernst Holler, Arne Kandulski, Daniela Hirsch, Anke Buttner, Wolf Rösler, Kai Hildner, Julia Winkler, Maike Büttner-Herold

**Affiliations:** 1 Institute of Pathology, University of Regensburg, Regensburg, Germany; 2 Institute of Pathology, University Medical Center Mainz, Mainz, Germany; 3 Institute of Pathology, Julius-Maximillians-University Würzburg, Würzburg, Germany; 4 Department of Nephropathology, Institute of Pathology, Friedrich-Alexander-University Erlangen-Nuremberg (FAU) and University Hospital, Krankenhausstr. 8-10, 91054 Erlangen, Germany; 5 Institute of Pathology, Friedrich-Alexander-University Erlangen-Nuremberg (FAU) and University Hospital, Erlangen, Germany; 6 Department of Internal Medicine II, University Hospital Wuerzburg, Würzburg, Germany; 7 Department of Internal Medicine III, University Medical Centre, Regensburg, Germany; 8 Department of Internal Medicine I, Gastroenterology, Hepatology, Endocrinology, Rheumatology and Infectious Diseases, University Hospital Regensburg, Regensburg, Germany; 9 School of Psychology, College of Life and Environmental Sciences, University of Birmingham, Birmingham, UK; 10 Department of Medicine 5, Friedrich-Alexander-University Erlangen-Nuremberg (FAU) and University Hospital, Erlangen, Germany; 11 Department of Medicine 1, Kussmaul-Campus for Medical Research and Translational Research Center, Friedrich-Alexander-Universität (FAU) Erlangen-Nürnberg, University Hospital Erlangen, Deutsches Zentrum Immuntherapie (DZI), Erlangen, Germany

**Keywords:** Round-Robin test, Acute Graft-versus-host disease, Colon biopsy, Interobserver agreement, Grading

## Abstract

Histomorpholgy is one of the mainstays of acute Graft-versus-host disease (GvHD) diagnosis. However, concerns about reproducibility and the most appropriate grading system question its usefulness. Our aim was to assess histomorphological parameters and previously reported grading systems for GvHD regarding reproducibility and validity. Moreover, we propose that sum scores, derived by combining separately scored morphological parameters into a total score, might provide a simplified but equally effective means to grade GvHD. A total of 123 colon biopsies were assessed across four pathologists for intestinal GvHD using a Round-Robin test and results were correlated with clinical findings. Interobserver reproducibility was high for histological parameters that were evaluated as indicators of acute GvHD. Published grading systems were moderately reproducible (ICC 0.679–0.769) while simplified sum scores, in comparison, showed better interrater reliability (ICC 0.818–0.896). All grading systems and sum scores were associated with clinical signs of GvHD and in part with therapy response and survival. However, they were not able to stratify patients according to the clinical severity of GvHD. In a hot-spot analysis 1 crypt apoptotic body (CAB) in 10 crypts was a reasonable cut-off value for minimal diagnostic criteria of GvHD. In conclusion, histology can contribute to the diagnosis of GvHD and is reproducible. Published grading systems are able to reflect clinical findings as are simplified sum scores, which showed improved reproducibility and might be easier to handle as they are based on adding up histological parameters rather than transferring histological findings into a separate grading system. Sum scores will have to be further tested in a prospective setting.

**Supplementary Information:**

The online version contains supplementary material available at 10.1007/s00428-023-03544-3.

## Introduction

Acute Graft-versus-host disease (aGvHD) is one of the most threatening complications of allogenic hematopoietic stem cell transplantation (alloHSCT). The gastrointestinal tract (GI) is a major target organ [25]. However, a widely accepted standard for histological GvHD reporting has not yet been established. This is reflected in the existence of numerous different grading systems that are applied in studies assessing histological findings of aGvHD, with Lerner grade being one of the most widely used [2, 6, 9, 12–14, 16–20, 24, 26, 28, 31, 33–35]. The lack of universally accepted standards hampers comparability of previous studies of aGvHD. Additionally, there have been reports of discrepancies when correlating histological and clinical findings [1, 28].

Moreover, as interobserver reproducibility is an issue, much effort has been made to standardize histopathological GvHD diagnoses [14, 31]. The latest modification of NIH categories for GvHD grading is strongly simplified including only “no”, “possible”, or “likely” GvHD [25, 31].

The present Round-Robin test aimed to improve reproducibility and standardisation of morphological changes of colorectal aGvHD. Several preexisting grading systems and newly generated sum scores were compared to identify the most robust and reproducible tool reflecting clinical findings.

## Material and methods

### Selection of patients, biopsies and clinical data

Inclusion criteria were a history of alloHSCT and colon biopsies taken 20 to 180 days after transplantation. Biospsies with signs of infection were excluded. Patients were randomly selected (Erlangen (n = 22), Mainz (n = 38), Regensburg (n = 51), and Würzburg (n = 12)). Age, sex, primary disease, days post transplant, GvHD-stage lower gastrointestinal tract (GI), overall Glucksberg grade (Supplemental Table 1 [11, 27]), response to steroid treatment (not applied/sensitive/ refractory/intolerant) and primary cause of death were retrieved from the MAGIC data base or clinical files (Supplemental Table 2). The overall Glucksberg grade is a combined value of clinical signs of GvHD in the skin, liver, upper and lower GI giving a grade of the clinical severity of GvHD (Supplemental Table 1). The GvHD stage lower GI is the respective value of the lower GI tract included in the Glucksberg grade, which stratifies the degree of GvHD according to the daily volume (< 500; 500–999; 100–1500; > 1500 ml/day) and frequency (< 3; 3–4; 5–7; > 7 episodes/day) of diarrhea and additional symptoms as severe pain or bloody stool [11]. The study was approved by the local Ethics Committee of the University Hospital Regensburg (No. 18–900-101).

**Table 1:** ≥75% agreement and results of consensus meeting

≥ 75% agreement present/absent	1^st^ Round	2^nd^ Round	Changes after consensus meeting
	Group1	Group1&2	
	N = 27	N = 123	
Crypt destruction*	88.5%	95.1%	semi-quantitative
Crypt loss*	/	89.4%	new parameter, semi-quantitative
Epithelial denudation*	88.9%	92.7%	inclusion of granulation tissue, semi-quantitative
Ulceration	96.3%	/	omitted
Architectural distortion	80.8%	85.8%	definition specified
Increased eosinophils	85.2%	95.9%	≥ 5/HPF
Increased neutrophils	88.9%	91.9%	≥ 5/HPF
≥ 75% agreement Grade/stage			
Sale	59.3%	60.2%	crypt loss & crypt destruction for grade 2 and 3
Melson	48.1%	56.1%	crypt loss & crypt destruction for grade 2 and 3
Lerner	70.4%	72.4%	focal < $50\%$ diffuse ≥ 50%
NIH	85.2%	82.1%	based on histology alone

^*^for comparability of the degree of agreement of the 1^st^ and 2^nd^ round, parameters are indicated as present or absent, meaning that in semi-quantitative parameters grade 0 was rated as absent and grade 1–2 as present

**Table 2:** Association of pathological findings and graduation with clinical findings

		Morphology				Published grading systems				Sum scores		
	Subgroup	CAB§	Crypt destruction§	Crypt loss§	Epithelial denudation§	Sale§	Melson§	Lerner§	NIH§	Sum score 1°	Sum score 2°	Sum score 3°
Overall Glucksberg	No/0 (n = 7)	0.5 0–0.5	0 0–0	0.25 0–0.5	0 0–0	0.5 0–0.5	0.5 0.25–0.75	0.5 0–0.5	0.5 0.25–0.75	1 0.5–1.25	1 0.5–1.25	1.25 0.75–2
	Low/1&2 (n = 33–34)	3.5* 0–22.25	0 0–2	1* 0–2	0 0–1	1.875* 0–3.75	2* 0–3.75	1.25* 0–4	1.25** 0–2	2.5* 0–5	3* 0–8	3.75** 0–9.75
	High/3&4 (n = 35–41)	6.125*** 0–34.5	1* 0–2	2** 0–2	0.25** 0–1	2.75** 0–4	3*** 0–4	2*** 0–4	2*** 0–2	3** 0–5	4** 0–8	5.5** 1–11
GvHD-stage lower GI	No/0 (n = 11)	0.5 0–1.75	0 0–0.25	0.25 0–2	0 0–0.25	0.5 0–2.5	0.75 0.25–2.5	0.5 0–1.5	0.5 0.25–1	1.25 0.5–3	1.25 0.5–3.5	1.5 0.75–4.75
	Low/1&2 (n = 44–47)	5.5*** 0–34.5	0.25* 0–2	0.875 0–2	0 0–1	2.25* 0–3.75	2.25** 0–3.75	1.75** 0–4	2*** 0–2	3* 0–5	3.375* 0–8	4.625** 0–11
	High/3&4 (n = 19–23)	6*** 0–17.25	1** 0–2	2** 0–2	0.75** 0–1	3** 0–4	3*** 0–4	2.5*** 0–4	2*** 0–2	3** 0–5	4.25** 0–7.75	5.5** 1–9
Steroid response	Not applied (n = 10–11)	0.875 0.5–17.75	0 0–2	0.25 0–2	0 0–1	0.5 0–3.5	0.75 0.25–3.5	0.75 0.25–3.75	0.75 0.25–2	1.25 1–5	1.25 1–7.25	2.375 1–8.75
	Responsive (n = 38–39)	4.625 0–34.5	0 0–2	1 0–2	0 0–1	2.25 0–3.75	2.25 0–3.75	1.5 0–4	1.75 0–2	3 0–5	3.625 0–8	5 0–11
	Refractory (n = 25–30)	6 0–17.75	0.75 0–2	2* 0–2	0.375* 0–1	2.875* 0–4	3* 0–4	2.25* 0–4	2* 0–2	3 0–5	4* 0–7.75	5 1–9.75
Survival	Alive (n = 30–32)	2.25 0–20	0 0–2	0.625 0–2	0 0–1	1 0–3.75	1.5 0–3.75	1 0–3.75	1.25 0–2	1.375 0–5	1.5 0–8	2.5 0–11
	NRM (n = 30–35)	5.75* 0–22	1 0–2	2 0–2	0.25 0–1	3 0–4	3* 0–4	2.25* 0–4	2* 0–2	3* 0–5	4.125* 0–7.75	5.5 1–9.75
	RM (n = 15)	7.75* 0–34.5	0.5 0–2	0.5 0–2	0.25 0–1	1.75 0–3.75	2 0.75–3.75	1.75 0–3.5	2 0.25–2	3 0.5–5	3.75 0.5–8	4.75 0.75–8.75

Data presented as median (upper row) and min–max (lower row); n indicates the number of biopsies analyzed; § mean values of all four observers; ° sum scores generated from the mean values of the respective parameters; CAB = crypt apoptotic bodies; NRM = non-relapse mortality; RM = relapse mortality; * p < 0.05; ** p < 0.01; *** p < 0.001; significances indicated by asterisks are compared to Glucksberg grade 0; GvHD-stage lower GI 0, Steroid response: not applied and Survival: alive. For statistical analyses, overall Glucksberg grades and GvHD-stages lower GI were divided into cases with no signs of GvHD (grade 0), with low-grade (grades 1&2) and high-grade (grade 3&4) findings

### Histomorphological assessment and consensus meeting

The Round-Robin test was performed in two rounds with a consensus meeting between them (Suppl. Fig. 1). In the 1st round, 27 biopsies (at least 5 stained sections) of 10 patients (= Group1) were assessed by 3 experienced pathologists (S.R-H., A.K. and M. B.-H.) and a pathology fellow well acquainted to GvHD (K.Hip.). The section with the most severe changes was preselected (by K.Hip.) for analyses. Sections were digitized and made accessible via a CaseCentre 2.9 (3DHISTECH, Budapest, Hungary) for online microscopy. Parameters assessed in the first round included: number of apoptoses as defined by Kreft et al. [14] in 10 neighboring crypts in the hot-spot, as suggested previously [10]; presence or absence (yes/no) of crypt destruction [14], architectural distortion, increase of eosinophilic and neutrophilic granulocytes, ulceration and epithelial denudation [14]. Grading was performed according to modified Lerner [14, 16], Sale [24], Melson [19] and NIH categories [31].

**Fig. 1:**
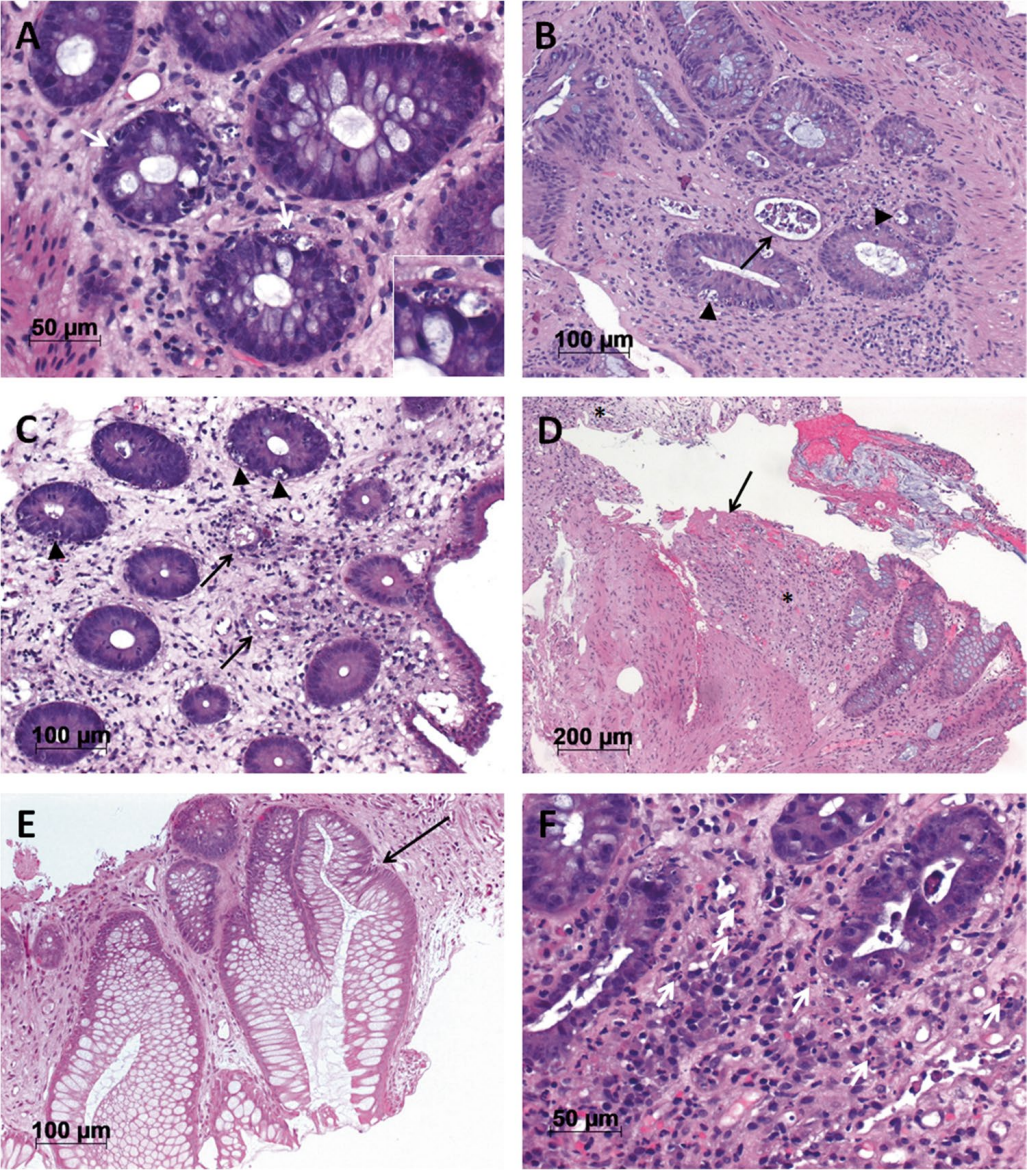
Histomorphological parameters evaluated by the 4 observers. (**A**) Crypts with several apoptotic bodies (CAB, arrows) with at least two fragments of karyorrhectic debris surrounded by a halo, enlarged in the inlay (H&E, 400x. original magnification (o.m.)). (**B**) Crypt destruction (arrow) with flattened epithelium of the crypt filled with cell debris. In the surrounding crypts several apoptotic bodies can be seen (arrow heads, H&E, 200 × o.m.). An alternative definition of crypt destruction according to Kreft et al. includes apoptotic destruction of ≥ 1/3 of the crypt epithelium with at least ½ of the diameter of a normal crypt retained [14] (**C**) Crypt loss (arrows) indicated by missing or strongly degenerated crypts (H&E, 200 × o.m.) not fulfilling the criteria of crypt destruction. CAB in surrounding crypts indicated by arrow heads. (**D**) Epithelial denudation with surface deposition of fibrin (arrow) and granulation tissue (asterisks, H&E, 100 × o.m.). (**E**) Crypt architectural distortion with a conspiciously branched crypt (arrow) next to a distorted crypt (H&E, 200 × o.m.). (**F**) Increased granulocytic infiltrate as exemplified by neutrophilic granulocytes (arrows) as defined by ≥ 5 granulocytes per 400 × high power field (H&E, 400 × o.m.)

After the 1st round a consensus meeting was held (K.Hip., S.R.-H., A.K., A.R., M.B.-H.) for standardization (Fig. 1):

- Crypt apoptotic bodies (CAB) [14]: number of apoptoses in 10 neighboring crypts in the hot-spot
- crypt destruction as defined [14]: 0 = none, 1 = individual, non-contiguous crypts, 2 = destruction of ≥ 2 neighboring crypts
- crypt loss, as defined by missing intact crypts without above-described signs of crypt-destruction as defined [14]: 0 = none, 1 = individual, non-contiguous crypts, 2 = loss of ≥ 2 neighboring crypts
- increase of eosinophilic or neutrophilic granulocytes (modified after [7]): ≥ 5 granulocytes in one high power field in the hot-spot excluding eschar in ulcer/erosion; 0 = no increase, 1 = increase
- architectural changes of the mucosa including at least one of the following: shortened crypts not reaching the lamina muscularis mucosae, distorted or branched crypts [23]; 0 = no or mild, 1 = moderate to severe architectural changes
- denudation/erosion [14] and/or granulation tissue; 0 = absent, 1 = present.

Ulceration was omitted and grading systems were adapted (Suppl. Table 3) to be independent of clinical findings.

In the second round (approximately one year later), Group1 was reassessed plus 96 additional biopsies (Group2). In Group2, which was correlated with clinical findings, only one colon specimen per time-point was included per patient.

### Sum scores generated from the histomorphological parameters and CAB count cut-offs

Sum scores from the histological findings (Suppl. Table 3) were generated as follows: Sum score 1 included a score of the mean CAB count of all four observers (CAB score: 0: no; 1: 1- < 5; 2: 5- < 10; 3: ≥ 10 CAB/10 continuous crypts) plus the mean score of crypt loss between the four observers. Sum score 2, in addition to these two parameters, included the mean values of crypt destruction and epithelial denudation. Sum score 3, in addition to the parameters included in sum score 2, also included the mean values of architectural distortion and of the increase of eosinophilic and neutrophilic granulocytes.

To assess the significance of CAB counts cut-off values for the mean CAB count were defined (mean CAB count < cut-off versus ≥ cut-off) and resulting groups were compared with clinical findings.

### Validation cohort

For the validation of sum scores 1 and 2, an independent cohort of 111 patients was analyzed by one patholgogist (A.K.) including cases from Mainz (n = 58) and Regensburg (n = 53). For each patient, the colon biopsy with the most severe signs of GvHD at the time-point was evaluated.

### Statistical analyses

Statistical analyses were performed using SPSS software (IBM Statistics SPSS 24). To compare the distribution of continuous and ordinal parameters between two or more groups Mann–Whitney and Kruskal–Wallis tests were chosen, respectively. For nominal parameters, cross-tabulation was applied using Chi^2^-testing and post-hoc testing as described by Beaseley et al. [3]. For correlation analyses, a Spearman test was performed. To assess the reproducibility between observers, inter-rater reliability (IRR) was quantified using Fleiss’s Kappa (for nominal parameters and ordinal parameters with no more than 5 possible values) and intra-class correlation (ICC, for all ordinal parameters, two-way model, agreement type, single unit), relying on the R statistical environment v. 4.0.3 (https://www.R-project.org/) and the irr package v. 0.84.1. (https://CRAN.R-project.org/package=irr). P < 0.05 was used to identify statistically significant findings.

## Results

### Patients´ cohort, 1st and 2nd round of the Round-Robin test

Patients’ characteristics are summarized in Supplemental Table 2. In the first round, separate analysis of the 27 biopsies (Group1) was performed by the four observers without prior discussion (Table 1). Thereafter, a consensus meeting was held to establish more concise definitions of histomorphological parameters (Fig. 1 and Suppl. Fig. 1). As a result, ulceration was omitted, crypt loss added and crypt destruction changed into a semi-quantitative parameter. Moreover, a cut-off of ≥ 5 cells per HPF was defined for the presence of increased eosinophils and neutrophils [7]. Additionally, some of the definitions for assigning a case to the grading systems were specified (Suppl. Table 3). In the second round, Group1 plus 96 newly selected biopsies (Group2) were assessed using the updated criteria. A consensus diagnosis was accepted when at least 3 of 4 observers assigned the same value to a respective biopsy. Results of this” ≥ 75% agreement “ before and after the Consensus meeting are summarized in Table 1. Improvement of” ≥ 75% agreement “ was mild to moderate looking at the histomorphological parameters, whereas the interobserver reproducibility of the grading systems was at best mildly improved. Best concordance was achieved for the most simplified NIH categories followed by the Lerner grade. Correlation of CAB counts between the observers was high in both rounds with only minimal improvement (Suppl. Table 4). As additional parameters of interrater reliability Fleiss‘ kappa values and intra-class correlation coefficient (ICC) were calculated (Suppl. Table 5). No improvement was seen in CAB counts, epithelial denudation or grading systems, whereas improvement was highest when assessing increased neutrophils and eosinophils. Sum scores generated from morphological parameters appeared to have better reproducibility than the prepublished grades.

### Histological findings, grading and clinical findings in Group2

**Fig. 2:**
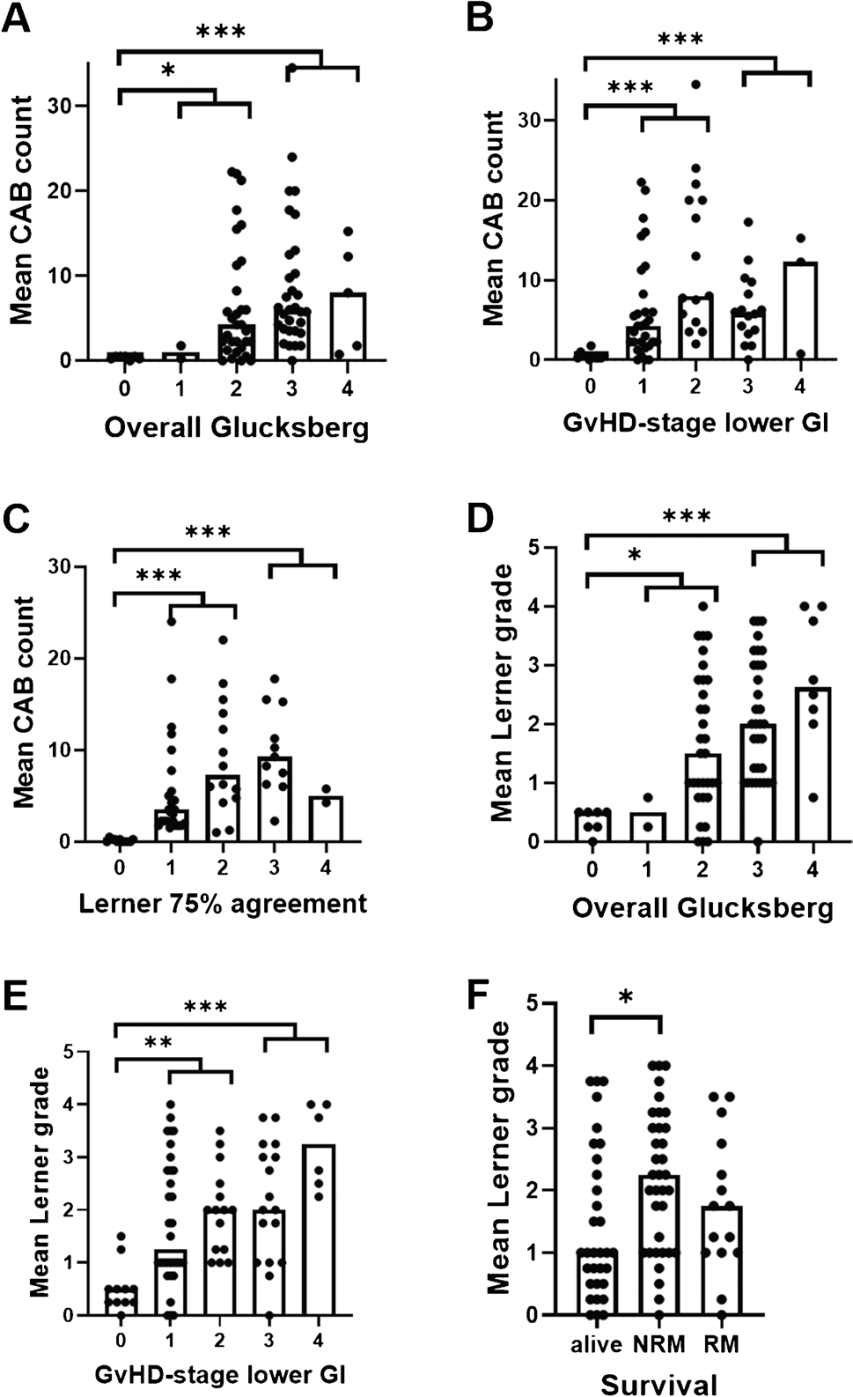
Association of morphological findings with clinical parameters. (**A**) Distribution of mean crypt apoptotic body (CAB) count related to clinical overall Glucksberg grades, showing a significant difference between no GvHD (grade 0) and low-grade (1&2) as well as high-grade (3&4) changes, resp.. (**B**) Mean CAB counts related to GvHD-stage lower GI with significant differences between no GvHD (grade 0) and low-grade (1&2) or high-grade (3&4) changes, resp.. (**C**) Comparison of the different Lerner grades (only cases with ≥ 75% agreement were included) in the distribution of mean CAB counts. Significant differences were seen between no signs of GvHD (Grade 0) and grades 1&2 and 3&4, resp.. (**D**) Mean Lerner grades increase with rising overall Glucksberg grades with a significant difference between no signs of GvHD (Grade 0) and low-grade (1&2) or high-grade changes (3&4), resp.. (**E**) Mean Lerner grades increased with GvHD-stage lower GI with a significant difference between stage 0 compared to 1&2 and 3&4, resp.. (**F**) Mean Lerner grades were additionally associated with survival, with higher Lerner grades in the NRM-group compared to living patients. Bars indicate the median. * p < 0.05; ** p < 0.01; *** p < 0.001

The mean values of CAB as assessed by the four observers in Group2 were significantly associated with overall Glucksberg grade (0 vs. 1&2, p = 0.01 and 0 versus 3&4, p < 0.001, Table 2, Fig. 2A), the GvHD-stage lower GI (0 vs. 1&2, p < 0.001 and 0 vs. 3&4, p < 0.001, Table 2, Fig. 2B), and the Lerner grades with ≥ 75% agreement (0 vs 1&2, p < 0.001; 0 vs 3&4, p < 0.001; Fig. 2C). Additionally, CAB counts were significantly higher in patients with non-relapse mortality (NRM, p = 0.021), but also with relapse mortality (RM, p = 0.012) when compared to living patients (Table 2). Other morphological parameters that reflect the clinical findings are summarized in Table 2. Increased eosinophilic and neutrophilic granulocytes and crypt architectural distortion were not associated with clinical findings (data not shown).

Regarding the grading systems (Table 2), Sale, Melson, Lerner (Fig. 2D-E), and NIH grades uniformly showed a significant association with overall Glucksberg grade (0 vs. 1&2: p = 0.044, 0.011, 0.014 and 0.006, resp. and 0 vs 3&4: p = 0.003, < 0.001, < 0.001 and < 0.001, resp.) and GvHD-stage lower GI (0 vs. 1&2: p = 0.047, 0.005, 0.002 and < 0.001, resp. and 0 vs 3&4: p = 0.001, < 0.001, < 0.001 and < 0.001, resp.). Moreover, higher Sale, Melson, Lerner, and NIH grades were associated with steroid refractoriness when compared to cases without application of steroids (p = 0.017, 0.010, 0.035 and 0.041, resp.). Higher Melson, Lerner (Fig. 2F), and NIH grades were also significantly associated with NRM when compared to living patients (p = 0.018, 0.025 and 0.029, resp.). None of the histological grading systems could differentiate between clinical low- and high-grade changes (Table 2).

### Sum scores as an alternative measure of grading GvHD and association with clinical findings

**Fig. 3:**
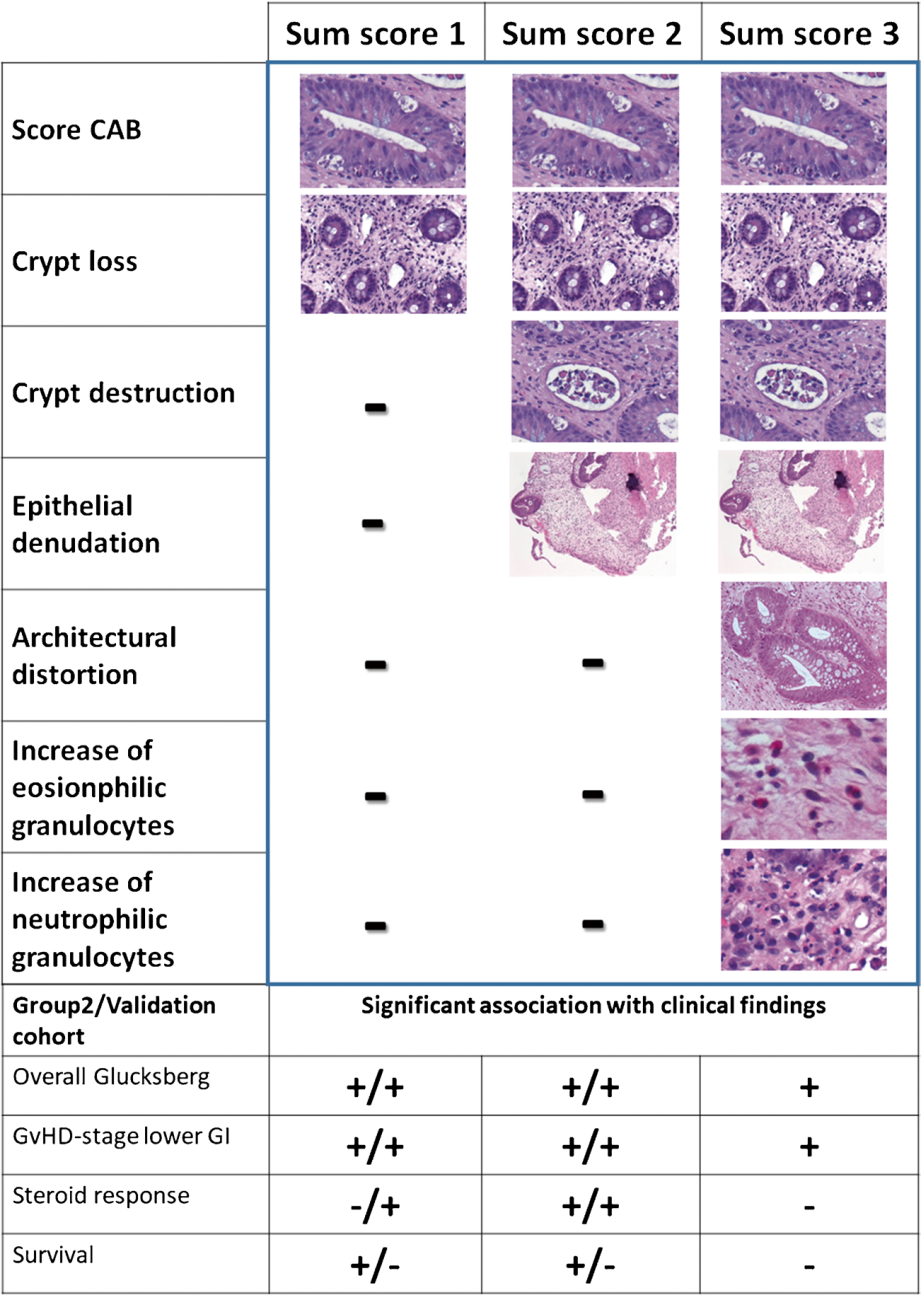
Sum scores—association with clinical findings. To test whether sum scores generated from the histological parameters might be useful for the grading of GvHD in colon biopsies, three different scores were generated and analyzed in the light of clinical findings. The most simplified sum score 1 included only a score of mean CAB counts and crypt loss, two parameters, which were frequently present in the biopsies and showed good association with clinical findings. Sum score 2 included parameters used in previous grading systems and sum score 3 included all parameters assessed in this study. Association with clinical findings was best in sum score 2 and better in sum score 1 than 3. For Group2 for the generation of sum scores results for the mean values of all 4 observerse are depicted with the CAB score being generated from the mean value of CAB, for the validation cohort results of one single pathologist (A.K.) are shown. In the lower part of the Figure significant associations of clinical findings for sum score 1 and 2 are shown for “group2/validation cohort” in comparison

As the transfer of histomorphological parameters into qualitative histological grading systems may give rise to misinterpretation or loss of information, we tested whether sum scores of histological parameters might better represent clinical findings (Fig. 3). The most simplified score included a score of mean CAB counts and crypt loss, both strongly associated with clinical findings (Table 2) and frequently observed in histological analysis of the cohort. Sum score 1 was significantly associated with overall Glucksberg grade (0 vs. 1&2, p = 0.024, 0 vs. 3&4, p = 0.002), GvHD-stage lower GI (0 vs. 1&2, p = 0.019, 0 vs. 3&4, p = 0.003), and survival (alive vs NRM, p = 0.013), but not response to therapy (Table 2). Sum score 2 included only parameters relevant for at least one pre-published grading systems (Sale, Melson, Lerner). Significant differences were observed for overall Glucksberg grade (0 vs. 1&2, p = 0.025, 0 vs. 3&4, p = 0.001), GvHD-stage lower GI (0 vs. 1&2, p = 0.013, 0 vs. 3&4, p = 0.001), survival (alive vs. NRM, p = 0.021), and steroid response (not applied vs. refractory, p = 0.032). For the most complex sum score 3 including all parameters assessed, no significant association with either steroid responsiveness nor survival was seen in contrast to overall Glucksberg (0 vs. 1&2, p = 0.009, 0 vs. 3&4, p = 0.001) and GvHD-stage lower GI (0 vs. 1&2, p = 0.005, 0 vs. 3&4, p = 0.001).

### Correlation between published GvHD grading systems, sum scores and clinical GvHD grading

Correlation analyses of established grading systems and sum scores showed a strong, positive association. The positive correlation with clinical parameters was moderate and within the same range regarding published grading systems and sum scores (Suppl. Table 6).

### Association of the validation cohort for sum scores with clinical signs of GvHD

To validate sum scores 1 and 2, an independent cohort of 111 cases was investigated by one pathologist (A.K.). Patients’ characteristics are summarized in Suppl. Table 7. In the validation cohort both analyzed sum scores (sum scores 1 and 2) were associated with clinical GvHD grading (Suppl. Table 8, Fig. 3). Both were able to differentiate between Glucksberg grades 0 vs. 3&4 and 1&2 vs. 3&4 (all p < 0.001) and GvHD-stage lower GI 0 vs. 1&2, 0 vs. 3&4, and 1&2 vs. 3&4 (sum score 1 p = 0.016, < 0.001, 0.009; sum score 2 p = 0.002, < 0.001, 0.002, resp.). Both sum scores were also different in cases, in which steroids were not applied vs. cases refractory to steroids (sum score 1 p = 0.019; sum score 2 p = 0.002). No association with mortality was observed.

### Relevance of CAB counts in reflecting clinical signs of GvHD

To analyze whether CAB counts, alone, could reflect clinical findings, cases were divided according to their CAB counts (Suppl. Table 9). Very low mean CAB counts of < 0.5 and/or < 1 were significantly associated with a lack of clinical signs of GvHD and no application of steroids. 100% of cases with no clinical signs of GvHD in both overall Glucksberg grade and GvHD-stage lower GI showed < 2 CAB. A cut-off of < 3 CAB was significantly associated with the absence of overall Glucksberg grade 3&4. Cut-off values of < 5–7 were significantly associated with patient survival in the follow-up. A cut-off of 6 CAB indicated approximately the median for cases with adverse clinical findings, ie. high-grade changes for overall Glucksberg and GvHD-stage lower GI, steroid refractoriness, and no relapse mortality, whereas 80–100% of the biopsies associated with favourable clinical findings had a CAB of < 6.

## Discussion

The present study aimed to assess the reproducibility and comparability of biopsy findings and grading of GvHD across pathologists at different HSCT centres. The diagnostic value of histology was determined by correlating histopathological characteristics and grading systems with clinical findings of GvHD. Finally, sum scores and different cut-offs for CAB counts were tested for their relevance in determining aGvHD.

The demographics of our cohort were within the range of previous studies [5, 6, 10, 14, 17, 18, 21, 26, 30, 32, 34]. In a first step morphological parameters and grading systems reported previously as diagnostic tools for GvHD reporting were tested for their reproducibility between pathologists. Before the first round of the Round Robin test, all observers familiarised themselves with histological criteria as defined earlier [14] without previous discussion. Agreement of ≥ 75% was high for dichotomized histomorphological parameters in the first round and further improved after consensus discussion. ≥ 75% agreement was much lower for the 3 to 5 tiered grading systems and improved only for Melson grading. Compared to a previous Round-Robin test [14] and a recent report assessing interrater reproducibility [26], our results were in the same range. Correlation between the observers in CAB counts was already high in the first round and no clear improvement was observed after the second round. These findings indicate that a relatively high comparability between different observers can be achieved just by studying the diagnostic criteria in the literature. A consensus meeting improves reproducibility in recognition and quantification of some morphological parameters, but appears to be less efficient in improving agreement in the application of grading systems.

In a next step the histological parameters were tested for their relevance as indicators of GvHD by comparing the mean values of all 4 observers with clinical signs of GvHD. Mean values were used, to reflect the ambiguities of GvHD reporting. CAB counts reflected overall Glucksberg grade, GvHD-stage lower GI, and survival, whereas they could not predict responsiveness to steroids. Only mean CAB counts and crypt loss were able to differentiate between no signs of GvHD and low-grade changes in overall Glucksberg grading and CAB and crypt destruction when looking at GvHD-stage lower GI. None of the parameters was able to discriminate between overall Glucksberg grade or GvHD-stage lower GI 1&2 and 3&4, i.e. to stratify low-grade and high-grade clinical GvHD findings. Myerson et al. proposed to subclassify Lerner grade 1 according to the numbers of CAB, which correlated with increased frequency of treatment [20], also arguing for the importance of apoptosis in the detection of low-grade GvHD. Crypt destruction, epithelial denudation, and crypt loss were all associated with severe clinical signs of aGvHD. Crypt loss and epithelial denudation, additionally, predicted refractoriness to steroids. In line with this observations, an association of severe crypt loss with higher stool volumes [6, 19], longer duration of diarrhea [6] and steroid refractoriness [19] has been reported before. Increased numbers of eosinophilic or neutrophilic granulocytes and architectural distortion were not significantly associated with clinical findings of GvHD in our cohort. Accordingly, eosinophilic counts did not support the diagnosis of colonic GvHD in previous reports [26, 30]. Increased neutrophilic granulocytes have been reported to be associated with inferior survival in GvHD of the upper GI [32], an association which we did not observe in the colon.

After evaluation of single morphological parameters, published grading systems based on these parameters were assessed for their association with clinical GvHD. All previously published [16, 19, 24, 31] histopathological grading systems were associated with clinical findings. Correlation between the grading systems was high, whereas correlation with clinical findings was only moderate. All grading systems could differentiate a group with no clinical signs of GvHD from low-grade or high-grade changes with regard to overall Glucksberg or GvHD-stage lower GI. No grading system could discern low- from high-grade clinical aGvHD. In line with this, a lack of correlation of low versus high histological grades with clinical GvHD grading has been reported [12]. Survival comparing no and mild histological signs of GvHD (4-tiered NIH categories) was the same in an earlier study. However, comparing no/mild and moderate/severe catergories showed improved survival in the former [26]. Moreover, reportedly severe histological damage (Lerner grading) was associated with inferior treatment response and survival compared to lower grades [9]. In contrast to our results, a modified Lerner grading system was able to discern GvHD of low and high severity with regard to volume and duration of diarrhea [6], whilst histological findings were unable to predict steroid response [6]. Sale et al. reported an association of high clinical stages and stool volume with positive results for GvHD in rectal biopsies [24]. Taken together, histological grading appears to efficiently reflect clinical GvHD, whereas it was of limited value for stratifying the severity of clinical findings. Previous findings [13] and our results also justify the widespread use of Lerner grade to report histological findings of GvHD for scientific purposes [9, 12, 14, 17, 18, 20] as it was significantly associated with all assessed clinical parameters and showed good reproducibility, whilst not including clinical parameters in its definition as opposed to NIH categories [31]. Underlining this conclusion, Lerner grading was also associated with GvHD-related death in a recent report [8].

Next, sum scores were tested as an alternative means of grading GvHD, as transfer into qualitatively defined scores carries the risk of misclassification. In line with this, IRR for the sum scores was better than for previously published grading systems. Results of sum score 3, not unexpectedly, indicated that an unselective increase of parameters does not necessarily improve the predictive value. Even sum score 2, including parameters used in previous grading systems, was only slightly superior to the very simple sum score 1, which included only CAB counts and crypt loss. The advantage of sum score 2, however, was its association with steroid refractoriness.

To validate our approach to apply sum scores 1 and 2, we analyzed an independent cohort of colon biopsies evaluated by a single pathologist. This approach better reflects the daily routine in the diagnosis of GvHD than using the mean values of 4 pathologists. Both sum scores were significantly associated with overall Glucksberg, GvHD-stage lower GI, and steroid response, supporting the use of sum scores. In contrast to the Round-Robin test, in the validation cohort a significant difference between low-grade and high-grade clinical findings could be observed for overall Glucksberg and GvHD-stage lower GI, maybe due to the fact that only the most severely affected biopsies were specifically chosen for analysis. In line with our approach, Farooq et al. tested the use of a sum score to grade colonic GvHD [8] and found an association with GvHD-related death in one of two analyzed cohorts.

Another important issue in the daily routine of diagnostic pathology is the cut-off of CAB counts to diagnose GvHD with certainty. Sauvestre et al. reported that in GvHD CAB count always exceeded 5 per biopsy [26]. Others [10, 17] suggested a cut-off of ≥ 7 CAB per 10 contiguous crypts. Moreover, it was suggested to classify ≤ 6 CAB/10 crypts as”indeterminate for GvHD “ as this group showed heterogeneous clinical findings [17]. Moreover, as minimal criteria of GvHD ≥ 1 CAB/biopsy piece [31] or ≥ 0.07 CAB per section [20] have been suggested, whereas others used ≤ 3 CAB/biopsy fragment as a cut-off for a negative histology [12]. In normal colon mucosa specimens any CAB were reported in only 20–25% of cases [5, 15]. In our cohort, < 1 CAB/10 contiguous crypts were significantly associated with negative overall Glucksberg grade, negative GvHD-stage lower GI, and no application of steroids. All cases with negative overall Glucksberg and negative GvHD-stage lower GI were included in the group of biopsies with < 2 CAB/10 crypts. Therefore, < 1 CAB/10 crypts appeared to be a relatively reliable cut-off value to identify cases without GvHD.

Shortcomings of our study are the retrospective nature and the fact that for correlation with clinical data in the Round-Robin test only one paraffin specimen per time-point and patient was investigated, therefore neglecting possible differences between different biopsy sites. The relatively low number of cases included for the clinical correlation may also have obscured the stratification of low- and high-grade findings. Any study based on histology after HSCT may face the problem of differentiating GvHD from mycophenolate mofetil (MMF)-colitis since the latter may mimic intestinal GvHD histologically [22, 33]. However, in the setting of solid organ transplantation, MMF-colitis is associated with GvHD-like histology in only a subset of cases [4, 29]. Moreover, apoptotic microabscesses (classified as crypt destruction by us) were reported to be absent in MMF-colitis [33], so that the differential diagnosis of MMF-colitis would be limited mainly to a subset of cases, which are treated with MMF and have low-grade GvHD. The significant association of histological and clinical signs of GvHD argues against MMF-colitis strongly confounding our results.

Taken together, our data indicate that relatively high concordance of grading aGvHD between pathologists can be achieved, when histological parameters are well defined and easily recognized, whereas reproducibility of the more complex and poorly defined grading systems is more difficult to obtain. As it stands, all previously published histopathological grading systems showed high correlations with each other and were able to reflect clinical findings in a significant manner. Histology appears to be helpful in confirming the diagnosis of aGvHD, whereas reliability was much worse in terms of the stratification of GvHD severity. Additionally, more simplified sum scores showed a slightly better reproducibility, retaining a comparable correlation to the clinical findings, a concept that we were able to reproduce in a validation cohort. A definite cut-off in CAB counts for the diagnosis of aGvHD of the colon does not exist, however, cases without clinical signs of GvHD were significantly associated with < 1CAB/10 crypts.

In conclusion, for the moment a combination of Lerner grading, based on morphology alone, and assignment of the NIH category proposed by the NIH Consensus development project [31], but in part dependent on clinical information, appears to be a pragmatic approach for the reporting of intestinal GvHD. In future, sum scores, after additional validation, might offer a simplified means of grading GvHD as they were slightly more reproducible across our team than previously published histological gradings and more straightforward to use as morphological parameters are simply added up and not transferred into a qualitative new grade. Finally, even if only very few CAB are present in a biopsy the possibility of GvHD should be considered as a diagnosis.

## Supplementary Information

Below is the link to the electronic supplementary material.

Supplemental Fig. 1 Overview of the analyzed colon biopsies (PNG 119 kb)

Supplementary file1 (TIF 18252 KB)

Supplementary file2 (DOCX 48.7 KB)

## Data Availability

The original contributions presented are included in the article and/or Supplementary material. Further inquiries can be directed to the corresponding author.

## Declarations

The study was approved by the local Ethics Committee of the University Hospital Regensburg (No. 18–900-101).

## References

[1] Abraham J, Janin A, Gornet JM, Peffault de Latour R, Robin M, Xhaard A, de Fontebrune FS, Mary JY, Allez M, Porcher R, Socie G (2014). Clinical severity scores in gastrointestinal graft-versus-host disease. Transplantation.

[2] Aslanian H, Chander B, Robert M, Cooper D, Proctor D, Seropian S, Jain D (2012). Prospective evaluation of acute graft-versus-host disease. Dig Dis Sci.

[3] Beasley TM, Schumacker RE (1995). Multiple Regression Approach to Analyzing Contingency Tables: Post Hoc and Planned Comparison Procedures. J Exp Educ.

[4] Calmet FH, Yarur AJ, Pukazhendhi G, Ahmad J, Bhamidimarri KR (2015). Endoscopic and histological features of mycophenolate mofetil colitis in patients after solid organ transplantation. Ann Gastroenterol.

[5] Cardona DM, Detweiler CJ, Shealy MJ, Sung AD, Wild DM, Poleski MH, Balmadrid BL, Cirrincione CT, Howell DN, Sullivan KM (2018). Use of the National Institutes of Health Consensus Guidelines Improves the Diagnostic Sensitivity of Gastrointestinal Graft-Versus-Host Disease. Arch Pathol Lab Med.

[6] da Costa LNG, Costa-Lima C, de Meirelles LR, Carvalho RB, Colella MP, Aranha FJP, Vigorito AC, De Paula EV (2018). Association between histopathological alterations and diarrhea severity in acute intestinal graft-versus-host disease. Medicine (Baltimore).

[7] Daneshpouy M, Socie G, Lemann M, Rivet J, Gluckman E, Janin A (2002). Activated eosinophils in upper gastrointestinal tract of patients with graft-versus-host disease. Blood.

[8] Farooq A, Gonzalez IA, Byrnes K, Jenkins SM, Hartley CP, Hagen CE (2022). Multi-institutional development and validation of a novel histologic grading system for colonic graft-versus-host disease. Mod Pathol.

[9] Ferrara JL, Harris AC, Greenson JK, Braun TM, Holler E, Teshima T, Levine JE, Choi SW, Huber E, Landfried K, Akashi K, Vander Lugt M, Reddy P, Chin A, Zhang Q, Hanash S, Paczesny S (2011). Regenerating islet-derived 3-alpha is a biomarker of gastrointestinal graft-versus-host disease. Blood.

[10] Gomez AJ, Arai S, Higgins JP, Kambham N (2016). Clinicopathologic Threshold of Acute Colorectal Graft-versus-Host Disease. Arch Pathol Lab Med.

[11] Harris AC, Young R, Devine S, Hogan WJ, Ayuk F, Bunworasate U, Chanswangphuwana C, Efebera YA, Holler E, Litzow M, Ordemann R, Qayed M, Renteria AS, Reshef R, Wolfl M, Chen YB, Goldstein S, Jagasia M, Locatelli F, Mielke S, Porter D, Schechter T, Shekhovtsova Z, Ferrara JL, Levine JE (2016). International, Multicenter Standardization of Acute Graft-versus-Host Disease Clinical Data Collection: A Report from the Mount Sinai Acute GVHD International Consortium. Biol Blood Marrow Transplant.

[12] Im JS, Abraham SC, Saliba RM, Rondon G, Ross WA, Rashid A, Shpall EJ, Popat U, Qazilbash MH, Hosing C, Oran B, Shah N, Tewari P, Nieto Y, Kebriaei P, Champlin RE, Alousi AM (2017). Histologic Grade 1 Is Associated With Increased Nonrelapsed Mortality in Lower Gastrointestinal Graft Versus Host Disease. Am J Surg Pathol.

[13] Kreft A, Hippe K, Wagner-Drouet EM, Ries I, Kandulski A, Buttner-Herold M, Neumann H, Weber D, Holler E, Schindeldecker M (2021). An investigation of the diagnostic, predictive, and prognostic impacts of three colonic biopsy grading systems for acute graft versus host disease. PLoS One.

[14] Kreft A, Mottok A, Mesteri I, Cardona DM, Janin A, Kuhl AA, Andrulis M, Brunner A, Shulman HM, Negri G, Tzankov A, Huber E, Gastrointestinal Pathology Group of the German-Austrian-Swiss Gv HDC (2015) Consensus diagnostic histopathological criteria for acute gastrointestinal graft versus host disease improve interobserver reproducibility. Virchows Arch 467:255-263. 10.1007/s00428-015-1803-y 10.1007/s00428-015-1803-y 26164839

[15] Lee FD (1993). Importance of apoptosis in the histopathology of drug related lesions in the large intestine. J Clin Pathol.

[16] Lerner KG, Kao GF, Storb R, Buckner CD, Clift RA, Thomas ED (1974). Histopathology of graft-vs.-host reaction (GvHR) in human recipients of marrow from HL-A-matched sibling donors. Transplant Proc.

[17] Lin J, Fan R, Zhao Z, Cummings OW, Chen S (2013). Is the presence of 6 or fewer crypt apoptotic bodies sufficient for diagnosis of graft versus host disease? A decade of experience at a single institution. Am J Surg Pathol.

[18] Ma C, Maluf HM, Liu TC (2015). Acute graft-versus-host disease is more prevalent and severe in the lower than the upper gastrointestinal tract. Hum Pathol.

[19] Melson J, Jakate S, Fung H, Arai S, Keshavarzian A (2007). Crypt loss is a marker of clinical severity of acute gastrointestinal graft-versus-host disease. Am J Hematol.

[20] Myerson D, Steinbach G, Gooley TA, Shulman HM (2017). Graft-versus-Host Disease of the Gut: A Histologic Activity Grading System and Validation. Biol Blood Marrow Transplant.

[21] Nguyen CV, Kastenberg DM, Choudhary C, Katz LC, DiMarino A, Palazzo JP (2008). Is single-cell apoptosis sufficient for the diagnosis of graft-versus-host disease in the colon?. Dig Dis Sci.

[22] Papadimitriou JC, Drachenberg CB, Beskow CO, Cangro C, Wiland A, Klassen D, Weir M, Bartlett S (2001). Graft-versus-host disease-like features in mycophenolate mofetil-related colitis. Transplant Proc.

[23] Riddell RH, Goldman H, Ransohoff DF, Appelman HD, Fenoglio CM, Haggitt RC, Ahren C, Correa P, Hamilton SR, Morson BC (1983). Dysplasia in inflammatory bowel disease: standardized classification with provisional clinical applications. Hum Pathol.

[24] Sale GE, Shulman HM, McDonald GB, Thomas ED (1979). Gastrointestinal graft-versus-host disease in man. A clinicopathologic study of the rectal biopsy. Am J Surg Pathol.

[25] Salomao M, Dorritie K, Mapara MY, Sepulveda A (2016). Histopathology of Graft-vs-Host Disease of Gastrointestinal Tract and Liver: An Update. Am J Clin Pathol.

[26] Sauvestre F, Belleannee G, Breal C, Mohr C, Fong HI, Cossin S, Tabrizi R, Milpied N, Vigouroux S, Goussot JF, Marty M (2018). Histologic analysis has a prognostical value in colorectal biopsies assessed for suspicion of graft-versus-host disease. Virchows Arch.

[27] Schoemans HM, Lee SJ, Ferrara JL, Wolff D, Levine JE, Schultz KR, Shaw BE, Flowers ME, Ruutu T, Greinix H, Holler E, Basak G, Duarte RF, Pavletic SZ, Party ETCW, the E-NIHCGTF (2018) EBMT-NIH-CIBMTR Task Force position statement on standardized terminology & guidance for graft-versus-host disease assessment. Bone Marrow Transplant 53:1401-1415. 10.1038/s41409-018-0204-7 10.1038/s41409-018-0204-7 PMC6786777 29872128

[28] Scott AP, Tey SK, Butler J, Kennedy GA (2018). Diagnostic Utility of Endoscopy and Biopsy in Suspected Acute Gastrointestinal Graft-versus-Host Disease after Hematopoietic Progenitor Cell Transplantation. Biol Blood Marrow Transplant.

[29] Selbst MK, Ahrens WA, Robert ME, Friedman A, Proctor DD, Jain D (2009). Spectrum of histologic changes in colonic biopsies in patients treated with mycophenolate mofetil. Mod Pathol.

[30] Shidham VB, Chang CC, Shidham G, Ghazala F, Lindholm PF, Kampalath B, George V, Komorowski R (2003). Colon biopsies for evaluation of acute graft-versus-host disease (A-GVHD) in allogeneic bone marrow transplant patients. BMC Gastroenterol.

[31] Shulman HM, Cardona DM, Greenson JK, Hingorani S, Horn T, Huber E, Kreft A, Longerich T, Morton T, Myerson D, Prieto VG, Rosenberg A, Treister N, Washington K, Ziemer M, Pavletic SZ, Lee SJ, Flowers ME, Schultz KR, Jagasia M, Martin PJ, Vogelsang GB, Kleiner DE (2015). NIH Consensus development project on criteria for clinical trials in chronic graft-versus-host disease: II. The 2014 Pathology Working Group Report. Biol Blood Marrow Transplant.

[32] Socie G, Mary JY, Lemann M, Daneshpouy M, Guardiola P, Meignin V, Ades L, Esperou H, Ribaud P, Devergie A, Gluckman E, Ameisen JC, Janin A (2004). Prognostic value of apoptotic cells and infiltrating neutrophils in graft-versus-host disease of the gastrointestinal tract in humans: TNF and Fas expression. Blood.

[33] Star KV, Ho VT, Wang HH, Odze RD (2013). Histologic features in colon biopsies can discriminate mycophenolate from GVHD-induced colitis. Am J Surg Pathol.

[34] Thompson B, Salzman D, Steinhauer J, Lazenby AJ, Wilcox CM (2006). Prospective endoscopic evaluation for gastrointestinal graft-versus-host disease: determination of the best diagnostic approach. Bone Marrow Transplant.

[35] Wild D, Sung AD, Cardona D, Cirricione C, Sullivan K, Detweiler C, Shealy M, Balmadrid B, Rowes KL, Chao N, Piryani S, Karimabad HM, Martin P, Poleski M (2016). The Diagnostic Yield of Site and Symptom-Based Biopsies for Acute Gastrointestinal Graft-Versus-Host Disease: A 5-Year Retrospective Review. Dig Dis Sci.

