# Supplementary figures and images for: Round-Robin test for the histological diagnosis of acute colonic Graft-versus-Host disease validating established histological criteria and grading systems
Source: Virchows Arch. 2023 May 11;483(1):47–58. doi: 10.1007/s00428-023-03544-3 (PMC10326090; doi:10.1007/s00428-023-03544-3)

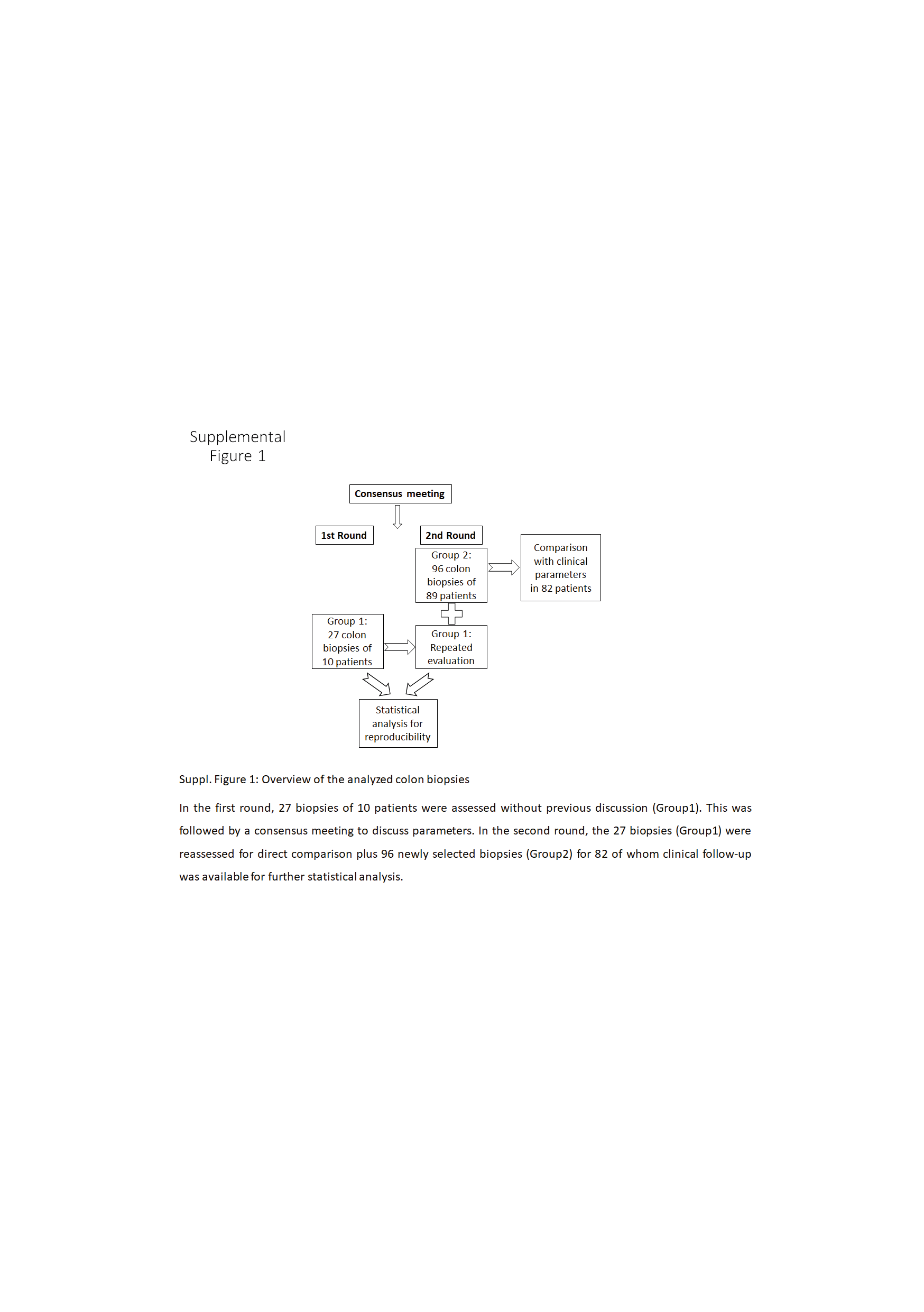

Supplement: Supplementary file 1 — Supplemental Fig. 1 Overview of the analyzed colon biopsies (PNG 119 kb) [file 428_2023_3544_Fig4_ESM.png]

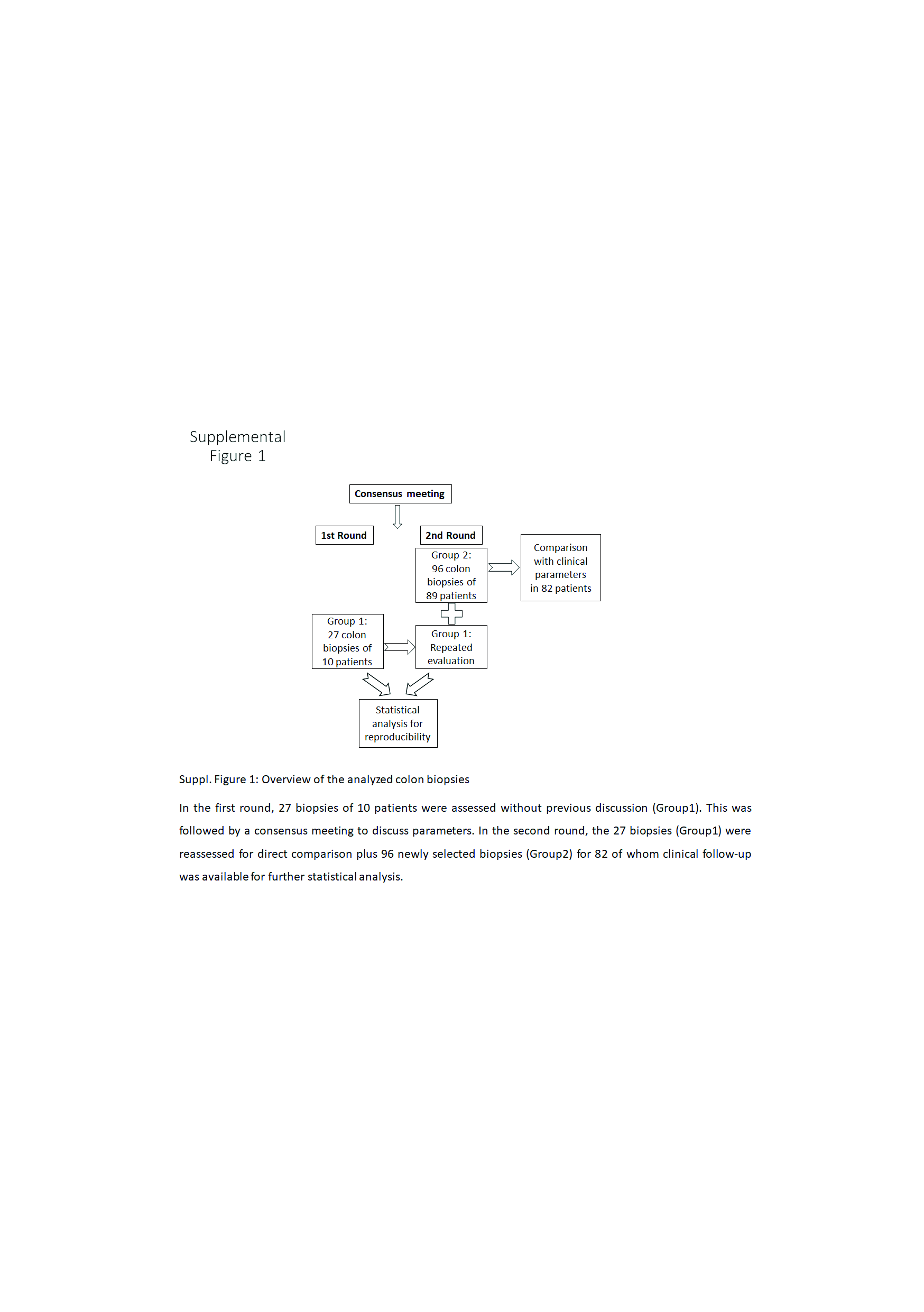

Supplement: Supplementary file 2 — Supplementary file1 (TIF 18252 KB) [file 428_2023_3544_MOESM1_ESM.tif]
