# Supplementary material for: Round-Robin test for the histological diagnosis of acute colonic Graft-versus-Host disease validating established histological criteria and grading systems
Source: Virchows Arch. 2023 May 11;483(1):47–58. doi: 10.1007/s00428-023-03544-3 (PMC10326090; doi:10.1007/s00428-023-03544-3)
Supplement: Supplementary file 3 — Supplementary file2 (DOCX 48.7 KB) [file 428_2023_3544_MOESM2_ESM.docx]

Supplemental Digital Content

Supplemental Table 1: Clinical graduation and staging of GvHD (after Harris et al.^11^)

**GvHD-stage organs**

| **Stage** | Skin | Liver | **Upper GI** | **lower GI** |
| --- | --- | --- | --- | --- |
| 0 | No (erythematous) GvHD rash | Bilirubin <2mg/dl | No or intermittent nausea, vomiting, or anorexia | diarrhea <500 ml/day or <3 episodes/day |
| 1 | Maculopapular rash <25% of body surface area (BSA) | Bilirubin 2-3 mg/dl | Persistent nausea, vomiting or anorexia with GvHD changes in upper GIT biopsy | 500-999 ml/day or 3-4 episodes/day |
| 2 | Maculopapular rash 25-50% of BSA | Bilirubin 3.1 -6 mg/dl |  | 1000-1500 ml/day or 5-7 episodes/day |
| 3 | Maculopapular rash >50% of BSA | Bilirubin 6.1-15 mg/dl |  | >1500 ml/day or >7 episodes/day |
| 4 | Generalized erythroderma (>50% BSA) with bullae and desquamation >5% BSA | Bilirubin >15 mg/dl |  | severe abdominal pain with or without ileus, or grossly bloody stool (regardless of stool volume) |

**Overall Glucksberg grade**

| **GvHD Grade** |  |
| --- | --- |
| **0 no** | No stage 1-4 of any organ |
| **I mild** | Stage 1-2 skin without liver, upper or lower GI involvement |
| **II moderate** | Stage 3 rash and/or stage 1 liver and/or stage 1 upper GI and/or stage 1 lower GI |
| **III severe** | Stage 2-3 liver and/or stage 2-3 lower GI, with stage 0-3 skin and/or stage 0-1 upper GI |
| **IV live threatening** | Stage 4 skin, liver, or lower GI involvement, with stage 0-1 upper GI |

GI: gastrointestinal tract

Supplemental Table 2: Demographics and clinical findings of the cohort

|  | 1st Round (Group1) | 2nd Round (Group2) |
| --- | --- | --- |
| Number of patients | 10 | 89 |
| Number of biopsies | 27§ | 96 |
| Age in years at transplantation; median (range)* | 53 (21-68) | 58 (15-75) |
| Sex (male:female)* | 8:2 | 56:33 |
| Days post HSCT; median (range)# | 39 (26-79), n=10 | 65 (20-180), n=95 |
| Overall Glucksberg grade (grade 0/1/2/3/4) | 1/0/4/2/3, n=10 | 7/2/32/33/8, n=82 |
| GvHD-stage lower GI (stage 0/1/2/3/4) | 1/0/4/3/2, n=10 | 11/32/15/17/6, n=81 |
| Steroid response (not applied/ sensitive/refractory/intolerant) | 4/1/5/0, n=10 | 11/39/30/0, n=80 |
| Primary disease* | AML (8)  MPN (1)  MDS (1) | AML (38)  MPN (8)  MDS (10)  MDS/MPN (4)  MM (6)  NHL (13)  ALL (9)  MPAL (1) |
| Leading cause of death* | Alive (5)  Relapse (1)  GvHD and infection (2)  Infection (1)  Renal (1) | Alive (31)  Relapse (12)  GvHD (8)  GvHD and infection (3)  Infection (17)  Cardiac (1)  Pulmonary (1)  Renal (1)  Hepatic (1)  Secondary malignancy (1)  Unknown (1)3 |

§ For 7 patients, more than one colon fraction at the analyzed time-point was included. * related to the analysed patients; # time-span as reported or calculated from the date of receipt of the biopsy/biopsies; n=number of submissions with seperate available information

AML: acute myeloid leukemia; MPN: myeloproliferative neoplasm; MDS: myelodysplastic syndrome; MDS/MPN: myelodysplastic/myeloproliferative neoplasia; MM: multiple myeloma; NHL: Non-Hodgkin lymphoma; ALL: acute lymphoblastic leukemia and MPAL: mixed phenotype acute leukemia.

Supplemental Table 3: Modified grading systems for aGvHD

| **Modified Lerner grade** | |
| --- | --- |
| Grade |  |
| 0 | Normal mucosa |
| 1 | Apoptosis of crypt or gland epithelia |
| 2 | Crypt or gland destruction* |
| 3 | Focal** mucosa denudation/erosion (including granulation tissue) |
| 4 | Diffuse*** mucosa denudation/erosion |
| **Modified Sale** | |
| Grade |  |
| 0 | None of the below mentioned findings |
| 1 | Crypt abscesses with necrotic or atypical epithelial cells, in vicinity of inflammatory infiltrate |
| 2 | Individual crypt loss/destruction# |
| 3 | Loss/destruction of two or more contiguous crypts |
| 4 | Total denudation of epithelium |
| **Modified Melson** | |
| Grade |  |
| 0 | No apoptotic cells, no crypt loss, no evidence of GI-GvHD |
| 1 | Apoptotic cells without crypt loss |
| 2 | Individual crypt loss or destruction only without contiguous areas of crypt loss and no crypt hyperplasia |
| 3 | Contiguous area of multiple crypt loss or destruction per high power field with or without presence of focal regenerative hyperplasia |
| 4 | Total crypt loss |
| **Modified NIH category** | |
| Category |  |
| Not | No evidence |
| Possible | Mild histomorphological changes compatible with GI-GvHD, but not sufficient for clear diagnosis |
| Likely | Typical histomorphological changes of GI-GvHD |
| **Sum scores** | |
| Sum score 1 | CAB counts (score 0-3) + crypt loss (score 0-2) |
| Sum score 2 | CAB counts (score 0-3) + crypt loss (score 0-2) + crypt destruction (score 0-2) + epithelial denudation (0 or 1) |
| Sum score 3 | CAB counts (score 0-3) + crypt loss (score 0-2) + crypt destruction (score 0-2) + epithelial denudation (0 or 1) + architectural distortion (0 or 1), increased eosinophils (0 or1) + increased neutrophils (0 or 1) |

*as defined by Kreft et al (Kreft, Virchows Arch, 2015, 467:255-263); **focal=less than 50% of mucosal surface, ***diffuse=50% or more of mucosal surface

#as defined by Kreft et al. but also missing crypts without visible signs of crypt undergoing destruction

CAB: crypt apoptotic bodies

Supplemental Table 4: Spearman correlation analysis of CAB counts between the observers

|  |  | Observer 1 | Observer 2 | Observer 3 |
| --- | --- | --- | --- | --- |
| Observer 2 | 1st Round (group1)  2nd Round (group1&2) | 0.895***  0.903*** | / | / |
| Observer 3 | 1st Round  2nd Round | 0.889***  0.903*** | 0.897***  0.853*** | / |
| Observer 4 | 1st Round  2nd Round | 0.840***  0.935*** | 0.891***  0.865*** | 0.902***  0.835*** |

***p<0.001

Supplemental Table 5: Interrater reliability - Fleiss’ kappa (for binary parameters) and ICC values

|  | 1^st^ Round (group1)  n=24-27 | | 2^nd^ Round (groups1&2)  n=112-123 | |
| --- | --- | --- | --- | --- |
|  | Fleiss’ kappa | ICC | Fleiss’ kappa | ICC |
| Crypt apoptotic bodies | / | 0.824 | / | 0.792 |
| Crypt destruction* | 0.651 | / | 0.787 | / |
| Crypt loss* | / | / | 0.576 | / |
| Epithelial denudation* | 0.719 | / | 0.681 | / |
| Architectural distortion* | 0.415 | / | 0.495 | / |
| Increased eosinophils* | 0.507 | / | 0.81 | / |
| Increased neutrophils* | 0.425 | / | 0.615 | / |
| Sale | / | 0.677 | / | 0.685 |
| Melson | / | 0.7 | / | 0.679 |
| Lerner | 0.404 | 0.83 | 0.469 | 0.769 |
| NIH | 0.629 | 0.835 | 0.549 | 0.711 |
| Sum score 1 | / | / | / | 0.818 |
| Sum score 2 | / | / | / | 0.888 |
| Sum score 3 | / | / | / | 0.896 |

* binary evaluation

Fleiss‘ kappa values (for binary parameters) and intra-class correlation coefficient (ICC, for ordinal parameters)

In the case of Lerner and NIH categories, Fleiss‘ kappa was computed in addition to ICC in order to be able to compare our results with previously published studies.

Supplemental Table 6: Spearman correlation analysis of histological and clinical grading

|  | Sale§ | Melson§ | Lerner§ | NIH§ | Glucksberg | Clinical stage GvHD intestine |
| --- | --- | --- | --- | --- | --- | --- |
| Melson§ | 0.983*** | / | / | / | / | / |
| Lerner§ | 0.920*** | 0.947*** | / | / | / | / |
| NIH§ | 0.729*** | 0.782*** | 0.810*** | / | / | / |
| Overall Glucksberg | 0.374** | 0.449*** | 0.470*** | 0.401*** | / | / |
| GvHD-stage lower GI | 0.385*** | 0.449*** | 0.481*** | 0.453*** | 0.877*** | / |
| Sum score 1§ | 0.850*** | 0.876*** | 0.830*** | 0.879*** | 0.363** | 0.393** |
| Sum score 2§ | 0.911*** | 0.933*** | 0.921*** | 0.881*** | 0.381** | 0.424*** |
| Sum score 3§ | 0.891*** | 0.919*** | 0.910*** | 0.847*** | 0.336** | 0.395*** |

*p<0.05; **p<0.01; ***p<0.001; § mean values of histological grading and sum scores are used for correlation analysis in Group2

Supplemental Table 7: Demographics and clinical findings of the validation cohort

|  | Validation cohort |
| --- | --- |
| Number of patients | 111 |
| Number of biopsies | 111 |
| Age in years at transplantation; median (range)* | 57 (18-74) |
| Sex (male:female)* | 69:42 |
| Days post HSCT; median (range) | 97 (20-197), n=111 |
| Overall Glucksberg grade (grade 0/1/2/3/4)§ | 10/4/44/40/13, n=111 |
| GvHD-stage lower GI (stage 0/1/2/3/4)§ | 14/42/22/25/8, n=111 |
| Steroid response (not applied/ sensitive/refractory/intolerant)§ | 15/64/24/2, n=105 |
| Primary disease* | AML (54)  MPN (8)  MDS (16)  MDS/MPN (3)  MM (6)  NHL (14)  ALL (6)  HD (2)  SAA (2) |
| Leading cause of death* | Alive 41  Relapse 12  GvHD 11  Infection 35  Cardio-vascular events 7  Second malignancy 2  Leukencephalopathy 2  Renal failure 1 |

* related to the analysed patients; n=number of biopsies with available information

AML: acute myeloid leukemia; MPN: myeloproliferative neoplasm; MDS: myelodysplastic syndrome; MDS/MPN: myelodysplastic/myeloproliferative neoplasia; MM: multiple myeloma; NHL: Non-Hodgkin lymphoma; ALL: acute lymphoblastic leukemia; HD: Hodgkin´s disease and SAA: severe aplastic anaemia.

Supplemental Table 8: Association of pathological findings and graduation with clinical findings in the validation cohort

|  |  | | **Sum scores** | | |
| --- | --- | --- | --- | --- | --- |
|  | | Subgroup | Sum score 1° | Sum score 2° |  |
| Overall Glucksberg | | No/0  (n=10) | 1  0-5 | 1.5  0-5 |  |
|  |  | Low/1&2 (n=48) | 2  0-5 | 3  0-8 |  |
|  |  | High/3&4 (n=53) | **4***/###**  **1-5** | **5***/###**  **2-8** |  |
| GvHD-stage lower GI | | 0  (n=14) | 2  0-3 | 2  0-3 |  |
|  |  | 1&2  (n=64) | **3***  **0-5** | **4****  **0-8** |  |
|  |  | 3&4  (n=33) | **4***/##**  **1-5** | **6***/##**  **2-8** |  |
| Steroid response | | Not applied (n=15) | 2  0-5 | 3  0-7 |  |
|  |  | Responsive (n=64) | 3  0-5 | 4  0-8 |  |
|  |  | Refractory  (n=24) | 4*  2-5 | **6****  **2-8** |  |
| Survival | | Alive  (n=41) | 3  0-5 | 3  0-8 |  |
|  |  | NRM  (n=59) | 3  0-5 | 4  0-8 |  |
|  |  | RM  (n=11) | 2  0-5 | 2  0-8 |  |

Data are presented as median (upper row) and min-max (lower row); n indicates the number of biopsies analyzed; NRM = non-relapse mortality; RM = relapse mortality; * or # p<0.05; ** or ## p<0.01; *** or ### p<0.001; significances indicated by asterisks are compared to Gluckberg grade 0; GvHD-stage lower GI 0 and Steroid response: not applied; significances indicated by hashes are compared to Glucksberg grade 1&2 and GvHD-stage lower GI 1&2.

Supplemental Table 9: Association of mean CAB counts in Group 2 with clinical findings for different cut-off values in cross tabulation

|  | Cut-off | <0.5 | <1 | <2 | <3 | <4 | <5 | <6 | <7 | <8 |
| --- | --- | --- | --- | --- | --- | --- | --- | --- | --- | --- |
| Overall Glucksberg | No/0 (n=7) | **42.9%*** | **100%***** | **100%***** | **100%**** | **100%*** | 100% | 100% | 100% | 100% |
|  | Low/1&2 (n=33) | 15.2% | 18.2% | 30.3% | 45.5% | 51.5% | 57.6% | 66.7% | 72.7% | 72.7% |
|  | High/3&4  (n=36) | 2.8% | **5.6%*** | 16.7% | **19.4%**** | 30.6% | 38.9% | 47.2% | 55.6% | 61.1% |
| GvHD-stage lower GI | 0  (n=11) | **45.5%**** | **81.8%***** | **100%***** | **100%***** | **100%***** | **100%**** | **100%*** | 100% | 100% |
|  | Low/1&2  (n=45) | 6.7% | **8.9%*** | **15.6**%** | 28.9% | 37.8% | 46.7% | 55.6% | 60% | 64.4% |
|  | High/3&4  (n=19) | 5.3% | 10.5% | 21.1% | 21.1% | 31.6% | 36.8% | 47.4% | 63.2% | 63.2% |
| Steroid response | Not applied (n=10) | 0% | **50%*** | 60% | 70% | 70% | 80% | 80% | 80% | 80% |
|  | Responsive (n=38) | 15.8% | 15.8% | 21.1% | 34.2% | 44.7% | 50% | 60.5% | 68.4% | 68.4% |
|  | Refractory  (n=26) | 7.7% | 11.5% | 26.9% | 26.9% | 34.6% | 42.3% | 50% | 57.7% | 65.4% |
| survival | Alive  (n=30) | 20% | 30% | 43.3% | **56.7%*** | 63.3% | **76.7%**** | **80%*** | **86.7%*** | 86.7% |
|  | NRM  (n=31) | 3.2% | 12.9% | 22.6% | 29% | 38.7% | 38.7% | 51.6% | 58.1% | 61.3% |
|  | RM  (n=15) | 13.3% | 13.3% | 20% | 20% | 26.7% | 33.3% | 40% | 46.7% | 53.3% |

*p<0.05; **p<0.01; ***p<0.001; percentages (%) indicate the percentage of cases below the cut-off in the respective group in Group2.

Percentages indicate the proportion of cases below the cut-off value in the respective group.
